# Supplementary material for: Time-periodic corner states from Floquet higher-order topology
Source: Nat Commun. 2022 Jan 10;13:11. doi: 10.1038/s41467-021-27552-6 (PMC8748824; doi:10.1038/s41467-021-27552-6)
Supplement: Supplementary file 2 — Supplementary Information [file 41467_2021_27552_MOESM2_ESM.pdf]

Supplementary Information for "Time-periodic corner states from Floquet higher-order topology"

Weiwei Zhu,[1,*] Haoran Xue,[2,*] Jiangbin Gong,[1,†] Yidong Chong,[2,3,‡] and Baile Zhang[2,3,§]

[1]*Department of Physics, National University of Singapore, Singapore 117542, Singapore*

[2]*Division of Physics and Applied Physics,*

School of Physical and Mathematical Sciences,

Nanyang Technological University, Singapore 637371, Singapore

[3]*Centre for Disruptive Photonic Technologies,*

Nanyang Technological University, Singapore 637371, Singapore

In this Supplementary Information, we present a number of additional results. In Sec. I, we provide a brief overview of Floquet theory and describe our system using a tight-binding model. In Sec. II, we present details of the design of the acoustic structure. In Sec. III, we show the spectra of finite structures with different parameters, showing the emergence of different topological phases. Sec. IV demonstrates that the properties of the corner modes are specific to Floquet systems, and discusses the topological invariants. In Sec. V, we use symmetry analysis to show that the AFHOTI has zero polarization and also to show that the topological nontrivial properties of the AFHOTI come from the singularities in the phase band of time evolution operator. In Sec. VI, we show the robustness of topological corner states. In Sec. VII, we discuss the coupling strength between two acoustic waveguides, including why the acoustic lattice can be mapped to tight-binding models, and how the coupling strength can be extracted from simulation data. Sec. VIII shows the measurement of acoustic couplings in experiment. Sec. IX shows the simulation results for field evolution in the three samples. In Sec. X, we show the experimental field evolution with bulk excitation for the three samples. Finally, in Sec. XI more experimental results are provided to show the unusual dynamical evolution in main text can be observed in a broad frequency range.

I. FLOQUET THEORY, TIGHT-BINDING MODEL AND PHASE DIAGRAM.

In this section, we provide an overview of Floquet theory and develop a theoretical model for the system shown in Fig. 2**a** of the main text.

For a periodically-driven Hamiltonian $H(t) = H(t + T)$ (where T is the driving period), we define the Floquet evolution operator (i.e., the time evolution operator in one period) as $U_F \equiv \mathcal{T}\exp\big[-i\int_{t_0}^{t_0+T} H(\tau)\,d\tau\big]$, where $\mathcal{T}$ is the time-ordering operator and t_0 is a reference time. We identify solutions $|\psi(t)\rangle = e^{-i\varepsilon t}|\phi(t)\rangle$, with $|\phi(t)\rangle = |\phi(t+T)\rangle$ and

$$U_F\,|\phi(t_0)\rangle = e^{-i\varepsilon T}|\phi(t_0)\rangle. \tag{S1}$$

The quasienergy ε is an angular variable with period $2\pi/T$.

Consider our system, which is shown schematically in Fig. S1**a**. It is a two-dimensional bipartite lattice with time-periodic modulation on the nearest-neighbour couplings. As illustrated in Fig. S1**a**, the modulation consists of four steps with equal duration $T/4$. In each step, each site only couples to one of its four neighbouring sites. The time-dependent

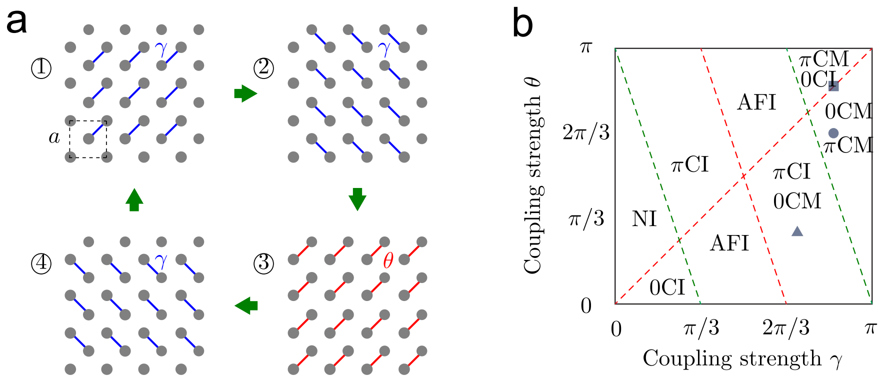

FIG. S1. Tight-binding model and phase diagram. **a**, The time-dependent tight-binding model proposed in this work. **b**, Phase diagram. Here NI, CI, AFI and CM denote normal insulator, Chern insulator, anomalous Floquet insulator and corner mode, respectively. Parameters for three experiment samples are marked by a square, circle and triangle. Red (green) dashes correspond to phase transition points where the bandgap at quasienergy zero (π/T) closes.

Bloch Hamiltonian is given by

$$H(\mathbf{k},t)=\sum_{m=1,2,3,4} C_m(t)\left(e^{i\mathbf{b}_m\cdot\mathbf{k}}\sigma^+ + \text{h.c.}\right), \tag{S2}$$

where $C_m(t)$ is set to θ during steps 1, 2, and 4 (modulo 4) step, and to γ during step 3 (modulo 4); $\sigma^\pm=(\sigma_x\pm i\sigma_y)/2$, where $\sigma_{x,y,z}$ are Pauli matrices; and the vectors $\mathbf{b}_m$ are given by $-\mathbf{b}_1=\mathbf{b}_3=(a/2,a/2)$ and $-\mathbf{b}_2=\mathbf{b}_4=(a/2,-a/2)$, where a is the lattice constant. We take $T=4$ and $a=1$ and express all variables in dimensionless units.

The lattice obeys a particle-hole symmetry $CH(k,t)C=-H^*(-k,t)$ and inversion symmetry $\mathcal{I}H(k,t)\mathcal{I}=H(-k,t)$, where $C=\sigma_z$ and $\mathcal{I}=\sigma_x$. With these symmetries, the lattice belongs to the D class, and can support a higher-order topological insulator (HOTI) phase associated with a Z_2 topological invariant[S1]. The particle-hole symmetry guarantees that the corner states come in pairs with quasienergy 0 or π/T, and the inversion symmetry further ensures that the paired corner states are localised at different edges. So a single pair of corner states cannot annihilate without bulk band gap closure[S1,S2].

Our model only contains two bands. The particle-hole symmetry constrains the band gap to existing only at quasienergy 0 or π/T. For each gap, the system can be a trivial insulator,

a first-order topological insulator with gapless edge states, or a second-order topological insulator with corner states. When sweeping the two coupling strength parameters θ and γ, various topological phases are found in both gaps. The phase diagram is shown in Fig. S1**b**. We will show the edge states and corner states in the spectra of finite structures in Section. III.

II. ACOUSTIC DESIGN

To realise the tight-binding model mentioned above, we design a 3D acoustic system to simulate the time-dependent 2D problem. Here, the z direction plays the role of time. Fig. S2 shows the designed acoustic structure which consists of two building blocks: waveguides and coupling channels. The waveguides act as lattice sites and the coupling channels enable coupling between neighbouring waveguides.

The configuration of coupling channels can be tuned along the z direction to realise the

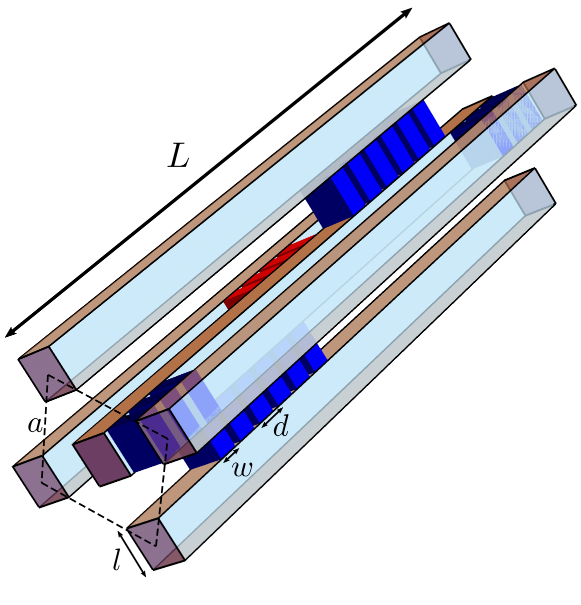

FIG. S2. Acoustic design. Here only one period with length L is shown. Black dashed lines indicate the unit cell. The structural parameters are: lattice constant $a = 20\sqrt{2}\,\text{mm}$, site length of the waveguides $l = 10\,\text{mm}$, width of coupling channels $w = 5\,\text{mm}$ and spacing between coupling channels $d = 7\,\text{mm}$.

time-dependent couplings of the tight-binding model[S3,S4]. Moreover, the coupling strength can be tuned by changing the number of couplings channels, as we will show in Section. VII. In Fig. S2, coupling channels that enable coupling strength θ and γ are colored in red and blue, respectively. With this flexible design, we are able to explore different topological phases given in Fig. S1**b**.

In order to facilitate the experimental measurement, each acoustic block (with length L that corresponds to a cycle of modulation) is designed to be composed of two half pieces fabricated separately. Each half piece has a length of $L/2$. Therefore, by stacking these $L/2$-long pieces, we are able to vary the length of the entire acoustic system as integer multiples of $L/2$.

III. EIGENSPECTRA OF FINITE STRUCTURES

In this section, we show the eigen spectra of finite structures with different coupling strength θ and γ, which indicate the existence of different topological phases and topological phase transitions.

Fig. S3**a** shows the results for $\theta = \gamma$, which lies at phase transition line as shown in phase diagram. When $0 < \gamma < \pi/4$, the system is a normal insulator; when $\pi/4 < \gamma < 3\pi/4$, it is an anomalous Floquet insulator that supports chiral edge states; when $3\pi/4 < \gamma < \pi$, it is a Floquet HOTI and supports π corner states.

Fig. S3**b** shows the result for $\theta = \pi/2$. By tuning γ, there are four different topological phases, normal insulator ($0 < \gamma < \pi/6$), Chern insulator in the π gap ($\pi/6 < \gamma < \pi/2$), Chern insulator in the π gap and 0 corner states ($\pi/2 < \gamma < 5\pi/6$) and anomalous Floquet HOTI ($5\pi/6 < \gamma < \pi$).

IV. TOPOLOGICAL CHARACTERISATION

In this section, we show that the corner states observed in our acoustic structure are specific to Floquet systems, and have no counterparts in static HOTIs.

In static systems, HOTIs are associated with either nonzero polarisation, or nonzero quadrupole moment (accompanied by zero polarisation). Since our system has only two bands, it cannot have nonzero quadrupole momentum, which requires at least four bands[S5].

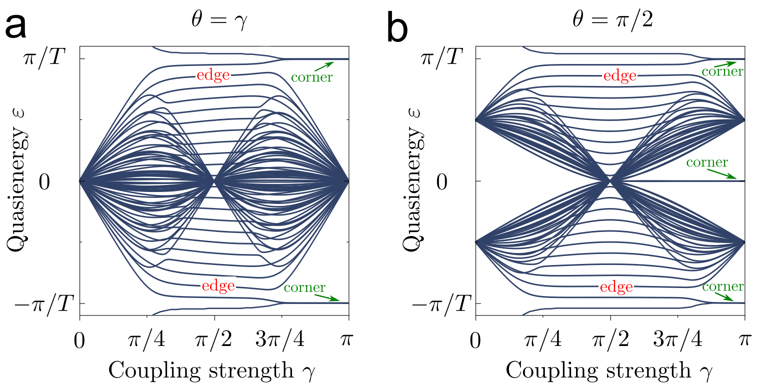

FIG. S3. Band diagrams for finite structures with 6 unit cells along x and y direction, with $\theta = \gamma$ (**a**) and $\theta = \pi/2$ (**b**).

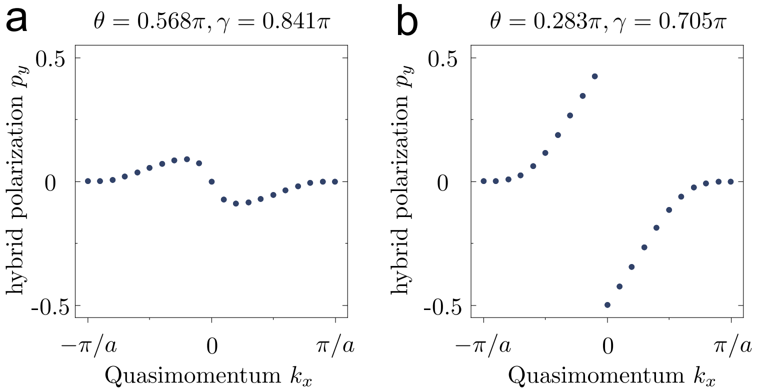

FIG. S4. Hybrid polarisation p_y as function of k_x using the parameters for second sample (**a**) and the parameters for third sample (**b**).

Next, we show that our system has zero polarisation by direct calculation. For the first sample ($\theta = \gamma = 0.841\pi$), the two bands are connected at the 0 gap. So we can treat it as a one band system whose polarisation is always zero. For the second sample ($\theta = 0.568\pi, \gamma = 0.841\pi$) and third sample ($\theta = 0.283\pi, \gamma = 0.705\pi$), we compute the hybrid polarisation

$$p_y(k_x) = -\frac{a}{2\pi}\int_{-\pi/a}^{\pi/a} dk_y A_y, \tag{S3}$$

where $A_y = -i\langle\phi(k_x,k_y)|\partial_{k_y}\phi(k_x,k_y)\rangle$ is the Berry connection and $|\phi(k_x,k_y)\rangle$ is the periodic part of the Bloch wavefunction. The polarisation can be obtained from the integral $P_y = \frac{a}{2\pi}\int_{-\pi/a}^{\pi/a} dk_x p_y(k_x)$. The hybrid polarisation for samples two and three are shown in Fig. S4. It is clear that the polarisation for sample two is zero ($P_y = 0$). Here we only show the polarisation along the y direction; similar results can be obtained for the x direction. The

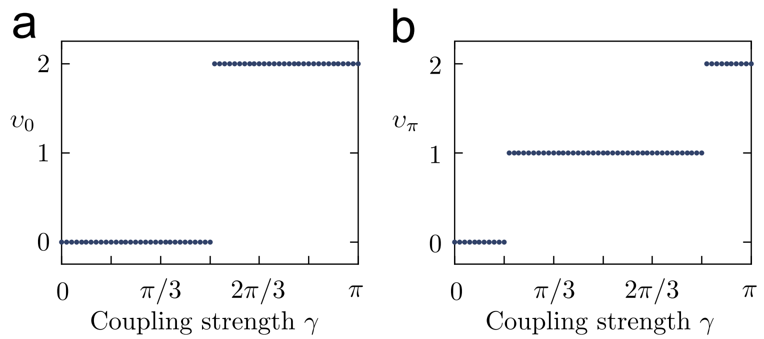

FIG. S5. The winding number with $\theta = \pi/2$ for the 0 gap ((**a**)) and π gap ((**b**)). Winding number is 1 for first-order topological insulator and 2 for second-order topological insulator.

winding of the hybrid polarisation provides the Chern number of the system. For sample three, the Chern number is 1, consistent with the fact that it supports 1D topological edge state in the π gap.

To understand the existence of the corner states in our system, we propose a dimensional reduction to a 1D Floquet Su-Schrieffer-Heeger model. Through bulk boundary correspondence, the number of edge states is determined by two winding numbers for the 0 gap and π gap respectively[S6]. For our system, with fixed $k_x = k_y = k$, the system reduces to the 1D Floquet SSH model. When the winding number is 1, the system has one edge state along each edge, meaning it supports chiral edge states. When the winding number is 2, the system has two counter-propagating edge states along each edge. In this case, the system has trivial first-order topology. However, now the system is a HOTI and supports corner states. The existence of the HOTI requires three conditions: (a) the system has chiral symmetry at special momentum values; (b) the winding number (0 gap or π gap) is nonzero; and (c) the system is gapped along the edges forming the corner.

We now describe the calculation of the winding numbers. With $k_x = k_y = k$, the Floquet evolution operator can be written with a symmetric formula,

$$U_T(k) = \sigma_z F^\dagger \sigma_z F, \tag{S4}$$

where

$$\begin{aligned} F &= e^{\frac{\theta}{2}\sigma_x} . e^{i\gamma(\cos(k)\sigma_x + \sin(k)\sigma_y)} . e^{i\frac{\gamma}{2}(\cos(2k)\sigma_x + \sin(2k)\sigma_y)} \\ &= \begin{pmatrix} a(k) & b(k) \\ c(k) & d(k) \end{pmatrix} . \end{aligned} \tag{S5}$$

The winding numbers for the 0 gap and π gap are defined as $\upsilon_0 = \upsilon(b)$ and $\upsilon_\pi = \upsilon(d)$. Fig. S5 shows the winding number for $\theta = \pi/2$. We notice that the 0 gap in Fig. S5**a** exhibits a topological phase transition from NI ($\upsilon_0 = 0$) to HOTI ($\upsilon_0 = 2$). And the π gap in Fig. S5**b** has a topological phase transition from NI ($\upsilon_\pi = 0$) to first order topological insulator ($\upsilon_\pi = 1$) and further to second order topological insulator ($\upsilon_\pi = 2$). These results are consistent with the results in Fig. S3**b**.

V. SYMMETRY ANALYSIS FOR ANOMALOUS FLOQUET HIGHER-ORDER TOPOLOGICAL INSULATOR

In this section, we use symmetry analysis to show that the AFHOTI has zero polarization and also show that the topological properties of the AFHOTI come from the singularities in the phase band of time evolution operator.

We first provide a general picture for the square lattice with inversion symmetry. The unit cell is shown in Fig. S6**a**. The Wyckoff positions ($1a$, $1b$, $1c$ and $1d$) can be obtained by $(x, y) = (-x, -y) + (ma, na)$ with $m, n \in Z$. The high symmetry momentum points (Γ, X, Y and M) are shown in Fig. S6**b** and can be obtained from $(k_x, k_y) = (-k_x, -k_y) + (2m\pi/a, 2n\pi/a)$ with $m, n \in Z$. According to the band representation theory, the elementary band representation can be obtained by putting s or p orbital to the Wyckoff position. All the atomic insulators can be represented as sum of these elementary band representations[S7]. We show all the elementary band representation of square lattice with inversion symmetry and their inversion symmetry eigenvalues at high symmetry momentum points in Table. I.

Next we focus on the AFHOTI with parameters $\theta = 0.568\pi$ and $\gamma = 0.841\pi$. The quasi-energy band is calculated by solving eigen equation,

$$U_T\,|\phi_n(k)\rangle = e^{-i\varepsilon_n T}|\phi_n(k)\rangle. \tag{S6}$$

where $U_T \equiv \mathcal{T}\exp\big[-i\int_0^T H(\tau)\,d\tau\big]$, and n numbers the bands. The symmetry eigenvalues at high symmetry momentum points can be directly obtained from

$$I|\phi_1(K)\rangle = \upsilon_K|\phi_1(K)\rangle \tag{S7}$$

For the AFHOTI, they are $(\upsilon_\Gamma, \upsilon_X, \upsilon_Y, \upsilon_M) = (+1, +1, +1, +1)$. Compared with Table. I, it corresponds to $s@q_{1a}$ which is s orbital at the center of unit cell. So the polarization of the

TABLE I. Inversion symmetry eigenvalues at HSMPs for eight elementary band representations of square lattice with inversion symmetry. Each band representation has either 0, 2 or 4 negative eigenvalues.

	Γ	X	Y	M
$s@q_{1a}$	+1	+1	+1	+1
$p@q_{1a}$	−1	−1	−1	−1
$s@q_{1b}$	+1	−1	+1	−1
$p@q_{1b}$	−1	+1	−1	+1
$s@q_{1c}$	+1	+1	−1	−1
$p@q_{1c}$	−1	−1	+1	+1
$s@q_{1d}$	+1	−1	−1	+1
$p@q_{1d}$	−1	+1	+1	−1

AFHOTI is zero in each direction. In a static system, the band with band representation $s@q_{1a}$ is topologically trivial. However, a Floquet system can support novel topological states without static counterparts. They are characterized by singularities in phase band[S8]. The phase band is obtained from eigen equation,

$$U_t(k)\,|\phi_n(k,t)\rangle = e^{-i\varphi(k,t)}|\phi_n(k,t)\rangle. \tag{S8}$$

where $U_t(k) \equiv \mathcal{T}\exp\left[-i\int_0^t H(\tau)\,d\tau\right]$. In our model, the singularities exist at high symmetry

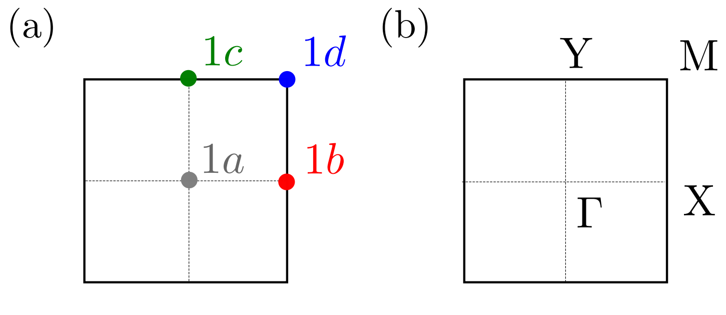

FIG. S6. **a**,The unit cell of a square lattice. Wyckoff positions of the square lattice with inversion symmetry are marked by $1a$, $1b$, $1c$ and $1d$. **b**, High symmetry momentum points in first Brillouin zone of the square lattice.

momentum points Γ and M. The phase bands for the AFHOTI at Γ and M points are plotted in Fig. S7**a**. At Γ point, there are two singularities with quasi-energy π and one singularity with quasi-energy 0. At M point, there are three singularities with quasi-energy 0. Only one of these three singularities are stable, because the middle one can annihilate with the left or right one. So in total there are two stable singularities for each quasi-energy π and 0. These even-number singularities in phase band of time evolution operator are the topological origin of topological corner modes we observed[S9,S10].

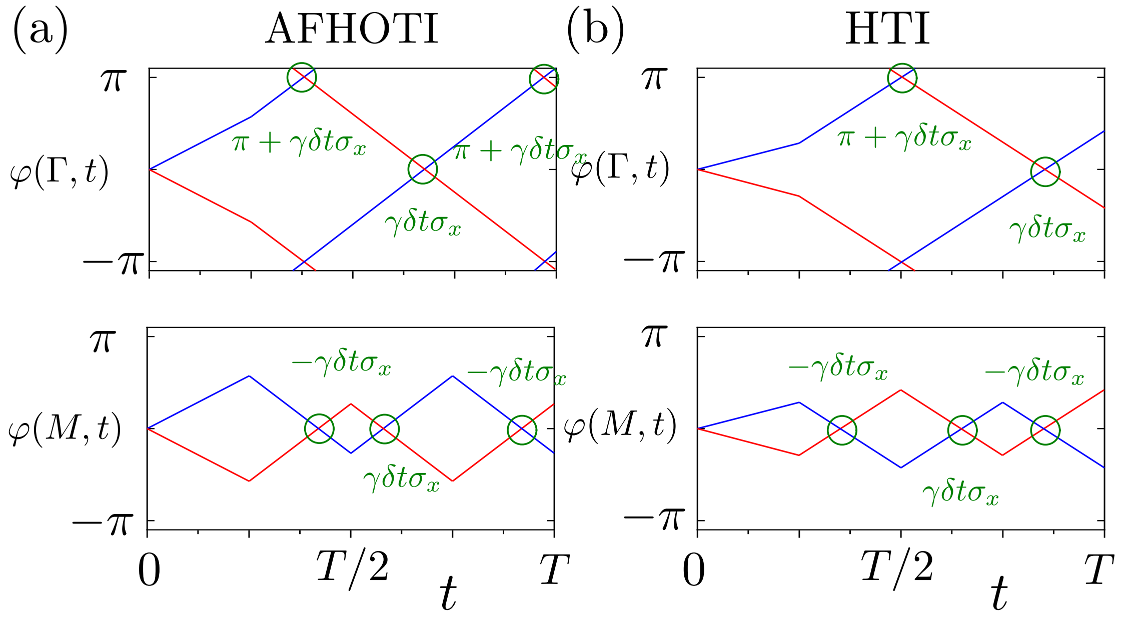

FIG. S7. Phase bands of time evolution operator at Γ and M points. (**a**) The results for second sample (AFHOTI) with parameters, $\theta = 0.568\pi$, $\gamma = 0.841\pi$. (**b**) The results for third sample (HTI) with parameters, $\theta = 0.283\pi$, $\gamma = 0.705\pi$. The inversion symmetry eigenvalue for the red (blue) bands is $-1(+1)$. The singularities are marked by green circles.

Fig. S7**b** shows the phase band for hybrid topological insulator (third sample) which supports chiral edge states in π gap and supports topological corner modes in 0 gap. We notice there are one stable singularity with quasi-energy π and two stable singularities with quasi-energy 0. So, the π gap is first order topological insulator and 0 gap is second-order topological insulator.

VI. ROBUSTNESS OF TOPOLOGICAL CORNER STATES AGAINST DISORDERS

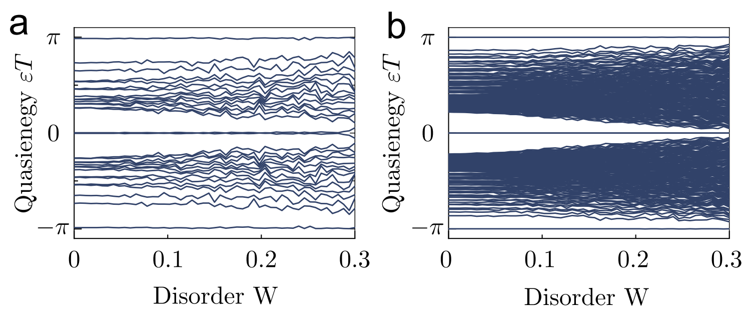

FIG. S8. Quasi-energy of finite structure as a function of disorder strength W. $\theta_0 = 0.568\pi, \gamma_0 = 0.841\pi$. **a** The size of the system are 4 unit cells along x and y direction as our experiment configuration. **b** The size of the system are 10 unit cells along each direction. The results are obtained from a tight-binding calculation.

In this section, we show the topological corner states are robust against disorders. As shown previously, our system obeys the particle-hole symmetry $CH(k,t)C = -H^*(-k,t)$ and inversion symmetry $IH(k,t)I = H(-k,t)$ where $C = \sigma_z$ and $I = \sigma_x$. Given two topological corner states, the particle-hole symmetry promises if one topological corner state has quasi-energy ε then another one must has quasi-energy $-\varepsilon+2n\pi/T$, where n is an integer. The inversion symmetry promises those two quasi-energies are equivalent or different by $2m\pi/T$ ($\varepsilon = -\varepsilon + 2n\pi/T + 2m\pi/T$), so that the topological corner modes can only have quasi-energy 0 or π/T in $(-\pi/T, \pi/T]$. Therefore, the quasi-energy of states #1 & #32 and #16 & #17 in Fig.3**a** cannot be moved unless the underlying symmetries are broken.

Practically, the topological protection can be verified by checking the robustness of topological states against disorders without breaking the symmetry or closing the band gap. We show in Fig. S8 the results for AFHOTI with disorder introduced to the couplings strength $\theta = \theta_0(1 + D)$ and $\gamma = \gamma_0(1 + D)$, where D is randomly distributed around $-W < D < W$. We notice that as the increase of disorder strength, the bulk band is broadening, but the topological corner modes are robust in quasi-energy at 0 and π/T, unless the disorders close the band gap eventually.

VII. COUPLINGS BETWEEN TWO ACOUSTIC WAVEGUIDES

In this section, we present the procedure for extracting the coupling strengths between two acoustic waveguides from numerical simulations. A schematic of two coupled waveguides is shown in Fig. S9**a**, with four ports marked 1, 2, 3 and 4. The coupling strength between these two waveguides can be tuned by changing the number of coupling channels. In simulations, we launch plane waves at port 1 with p_i and then determined the total acoustic pressure at the four ports (p_1, p_2, p_3, p_4). The scattering parameters at the four ports are $s_{11} = (p_1 - p_i)/p_i$, $s_{12} = p_2/p_i$, $s_{13} = p_3/p_i$ and $s_{14} = p_4/p_i$. The total reflection to port 1 and port 2 is $S_{11} + S_{12} = |s_{11}|^2 + |s_{12}|^2$.

Fig. S9**b** shows the simulation results for different numbers of coupling channels. We notice the total reflection is very small in the frequency range 6 kHz to 10 kHz, which means we can treat the two waveguides as a tight-binding model like $[p_o^3,\ p_o^4]^T = \exp(i\theta\sigma_x)\,[p_i^1,\ p_i^2]^T$. So we have $\tan|\theta| = |p_4/p_3|$ and $\theta = |\theta|$ $(\theta = \pi - |\theta|)$ when $p_3/p_i > 0$ $(p_3/p_i < 0)$. The coupling strengths for different numbers of coupling channels are shown in Fig. S9**c**. At 8 kHz, the coupling strengths for 4, 8, 10, 12 channels are 0.283π, 0.568π, 0.705π and 0.841π, respectively. Although we only consider the frequency 8 kHz in main text, the topological

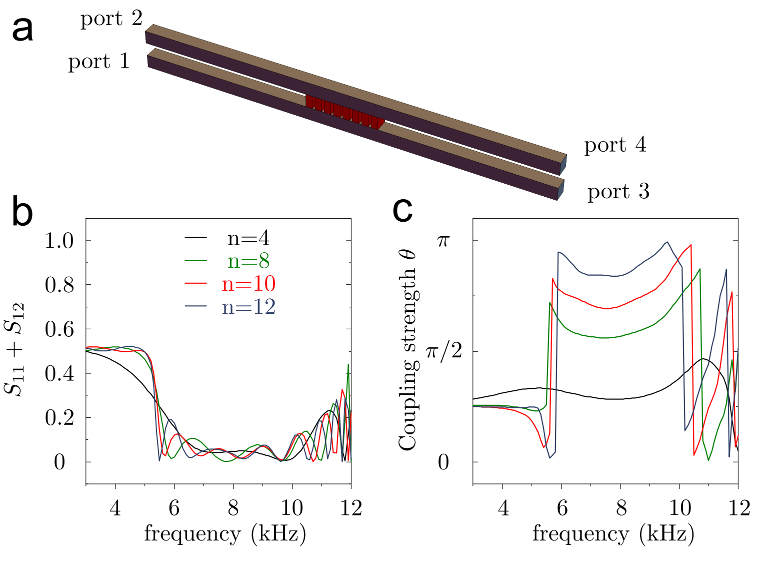

FIG. S9. Coupling between acoustic waveguides. **a**, Schematic diagram for two waveguides coupled by 8 coupling channels. **b**, The back scattering for coupled waveguides with n coupling channels. **c**, The extracted coupling strength for n coupling channels. At 8000 Hz, the coupling strength for 4, 8, 10, 12 channels is 0.283π, 0.568π, 0.705π and 0.841π, respectively.

states can be observed in a large frequency range 6 kHz to 10 kHz.

VIII. EXPERIMENTAL MEASUREMENT OF COUPLINGS BETWEEN TWO ACOUSTIC WAVEGUIDES

In this section, we measure the coupling strength in experiment. We first show the experimental setup. The schematic diagram of two coupled waveguide is shown in Fig. S10**a**. On the left, we set up one round hole to place speaker. On the right, we set up two round holes to place microphones. Besides, we also set up many rectangular holes on both waveguides, which is used to decrease the reflection from boundary. To measure the couplings strength, we also need one reference waveguide as shown in Fig. S10**b**.

The measured signal is p_1 for microphone 1 and p_2 for microphone 2. The measured signal is p_r for reference microphone. The couplings strength can be obtained from $\theta = \arccos(\pm|p_1|/\sqrt{|p_1|^2 + |p_2|^2})$. $+$ or $-$ is determined by the phase difference between p_1 and p_r. For 4 couplings blocks, $+$ is chosen due to that p_1 and p_r are in phase as shown in Fig. S11**a**. For 8, 10 and 12 couplings blocks, $-$ is chosen due to that p_1 and p_r are out

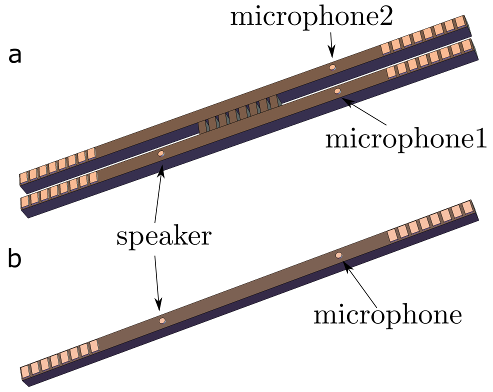

FIG. S10. Experimental setup of the measurement of couplings between two acoustic waveguides. **a**, The two coupled waveguides. The speaker is placed in the round hole on the left, and two microphones are placed in the round holes on the right. The measured signals are p_1 and p_2 respectively. **b**, The reference waveguide. The speaker is placed in the round hole on the left, and the microphone is placed in the round hole on the right. The measured signal is p_r. The rectangular holes are designed to decrease reflection from boundaries.

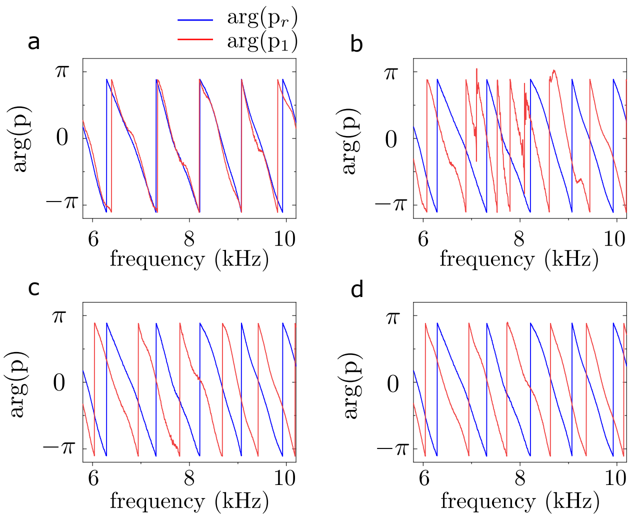

FIG. S11. Measured phase. The blue lines are phase for p_r and the red lines are phase for p_1. **a**, **b**, **c**, **d** are the results for 4, 8, 10, 12 coupling blocks.

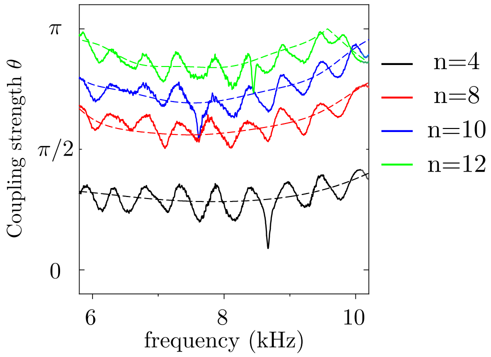

FIG. S12. Measured coupling strength for 4, 8, 10 , 12 coupling blocks. The measured coupling strengths (solid lines) are consistent with numerical simulation (dashed lines).

phase as shown in Fig. S11**bcd**. The extracted coupling strengths are shown in Fig. S12. We notice the measured coupling strengths are consistent with the numerical results. The oscillations come from the reflection from the boundary.

IX. SIMULATION RESULTS OF FIELD EVOLUTION FOR THREE SAMPLES.

In this section, we show simulated field evolution results for the three samples. The source is chosen to excite the system at one site, marked by the green arrow in Fig. S13. Fig. S13**a**

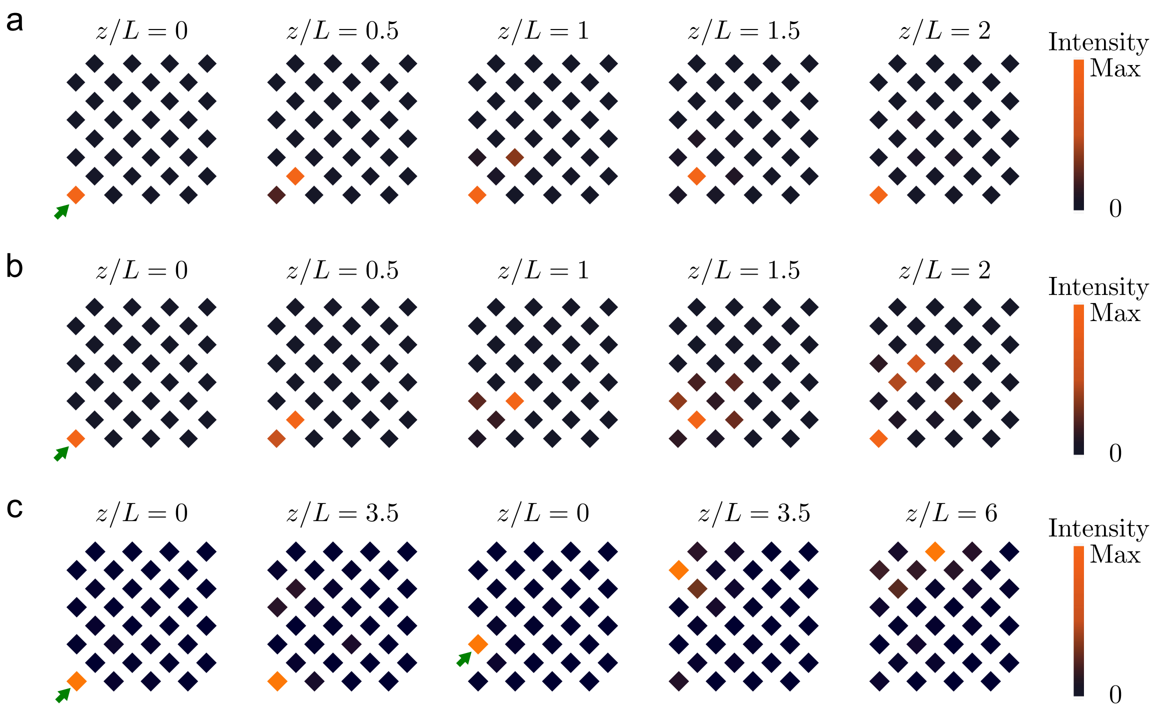

FIG. S13. Field evolution for three samples from COMSOL simulation. **a-c** are the results for three samples respectively. The source is marked by green arrow.

shows the results for sample one, which supports π mode. The field oscillates between the two sublattices. Fig. S13**b** shows the results for sample two, which simultaneously supports 0 and π modes. The field shows double-period oscillation. Fig. S13**c** shows the results for sample three, which simultaneously supports corner states and topological edge states. With different excitations, the field can be localised at the corner or propagate along the edge. Moreover, the edge states propagate unidirectionally and can go through corners without backscattering (see Fig. S13**c**), demonstrating their topological nature. These results are consistent with the experimental results presented in the main text.

X. EXPERIMENTAL RESULTS OF EVOLUTION PROFILE FOR BULK EXCITATION

In this section, we show the experimental field evolution results with bulk excitation for the three samples. The source is putted at one site as shown in first column of Fig. S14. Different from the corner excitation, the field for bulk excitation is extended over the bulk

rather than beling localized.

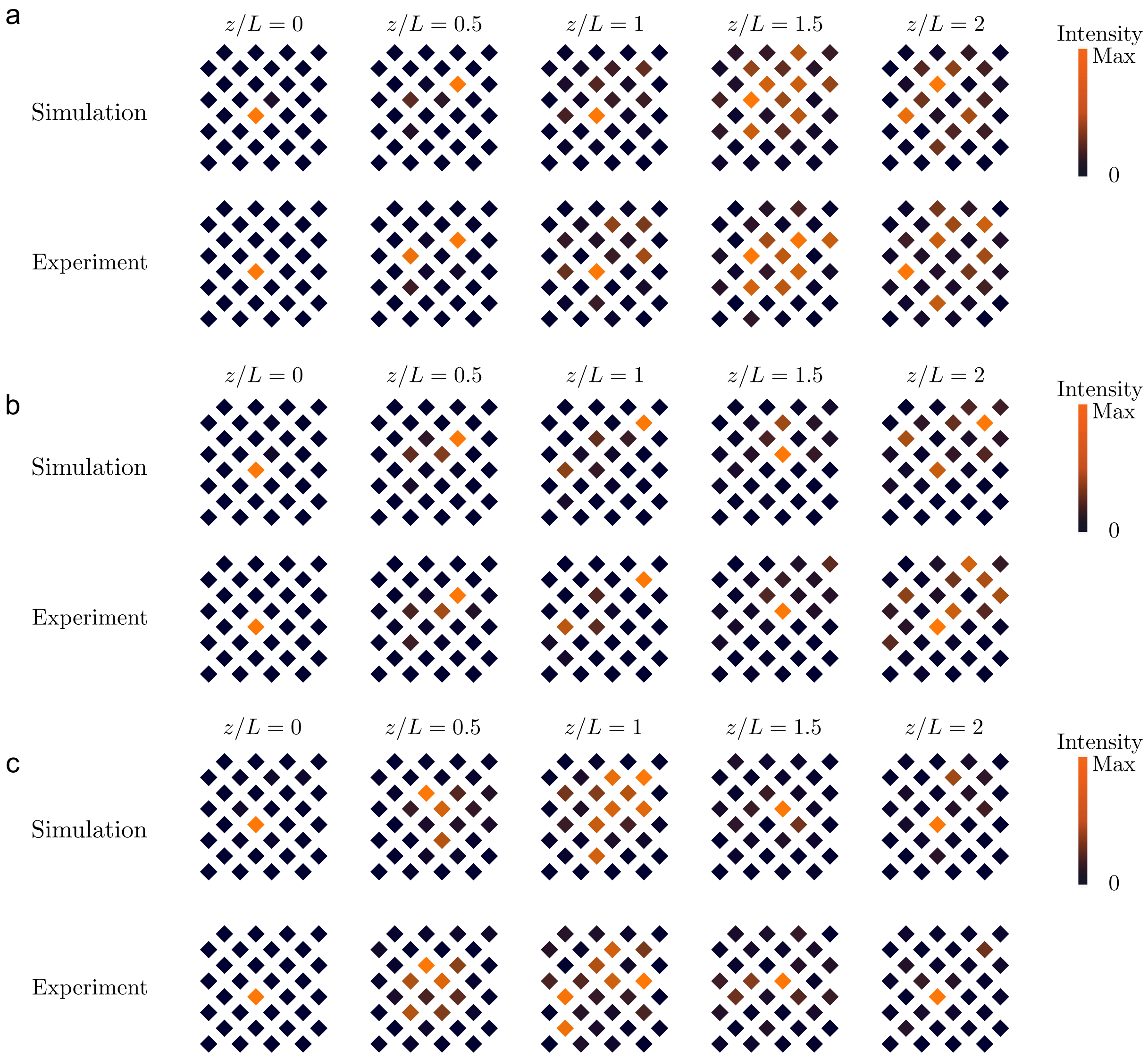

FIG. S14. Field evolution from simulation and experiment for bulk excitation. **a-c** The results for sample 1, sample 2 and sample 3, respectively. Fives columns are results at $z/L = 0$, $z/L = 0.5$, $z/L = 1.0$, $z/L = 1.5$ and $z/L = 2.0$, respectively.

XI. MORE EXPERIMENT RESULTS

In this part, we provide more experiment results to show the unusual dynamic evolution of corner states can be observed in a broad frequency range from 7500 Hz to 8300 Hz.

Fig.S15 shows the results for first sample which supports π corner states. We choose three frequencies 7500 Hz, 8000 Hz and 8300 Hz. At those frequencies, we all observe the π corner states are oscillating between two sublattices. This phenomena can be observed in frequency range from 7500 Hz t0 8300 Hz. Fig.S16 shows the results for second sample which simultaneously supports 0 and π corner states. We notice the field come back to corner sites at $z/L = 2.0$ which corresponding to the period doubling. The phenomena shown in Fig.3(d) can be observed in a large frequency range from 7500 Hz t0 8300 Hz.

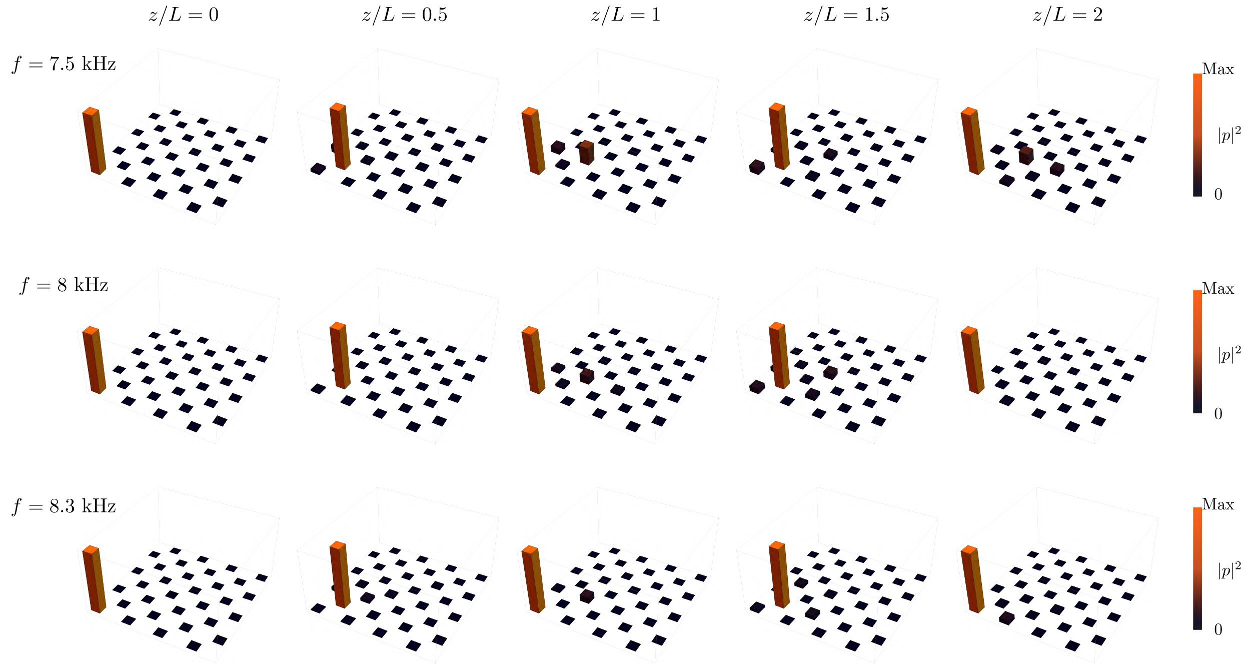

FIG. S15. Measured acoustic pressure for first sample which supports π corner mode. Three rows are the results for different frequency 7500 Hz, 8000 Hz and 8300 Hz. Fives columns are results at $z/L = 0$, $z/L = 0.5$, $z/L = 1.0$, $z/L = 1.5$ and $z/L = 2.0$, respectively.

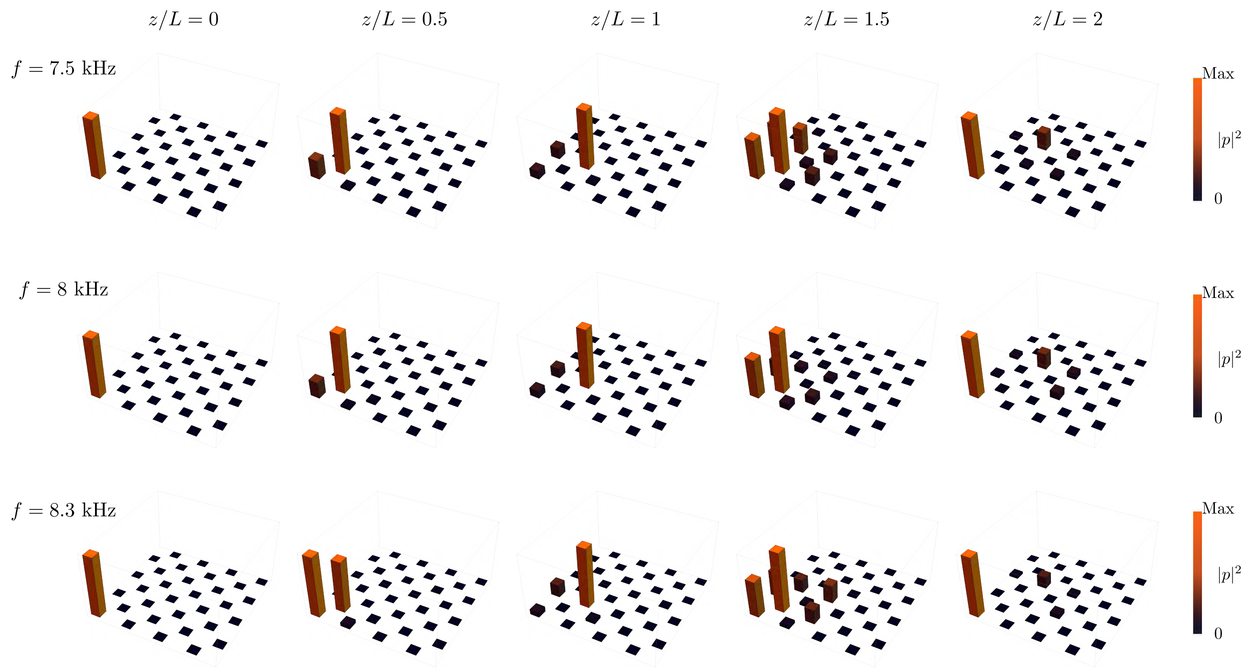

FIG. S16. Measured acoustic pressure for second sample which simultaneously supports 0 and π corner mode. Three rows are the results for different frequency 7500 Hz, 8000 Hz and 8300 Hz. Fives columns are results at $z/L = 0$, $z/L = 0.5$, $z/L = 1.0$, $z/L = 1.5$ and $z/L = 2.0$, respectively.

* These authors contribute equally.

† phygj@nus.edu.sg

‡ yidong@ntu.edu.sg

§ blzhang@ntu.edu.sg

[S1] E. Khalaf, Higher-order topological insulators and superconductors protected by inversion symmetry, Phys. Rev. B **97**, 205136 (2018).

[S2] W. W. Zhu, Y. D. Chong, and J. B. Gong, Floquet higher order topological insulator in a periodically driven bipartite lattice, Phys. Rev. B **103**, L041402 (2021).

[S3] Y. X. Shen, Y. G. Peng, X. C. Chen, D. G. Zhao, and X. F. Zhu, Observation of low-loss broadband supermode propagation in coupled acoustic waveguide complex, Sci. Rep. **7**, 1 (2017).

[S4] M. Moleron, C. Faure, S. Felix, V. Pagneux, and O. Richoux, Discrete propagation of trapped modes in acoustic waveguide arrays, Phys. Rev. B **99**, 201404 (2019).

[S5] W. A. Benalcazar, B. A. Bernevig, and T. L. Hughes, Quantized electric multipole insulators, Science **357**, 61 (2017).

[S6] J. K. Asbóth, B. Tarasinski, and P. Delplace, Chiral symmetry and bulk-boundary correspondence in periodically driven one-dimensional systems, Phys. Rev. B **90**, 125143 (2014).

[S7] J. Cano, and B. Bradlyn, Band Representations and Topological Quantum Chemistry, Annu. Rev. Condens. Matter Phys. **12**, 225 (2021).

[S8] F. Nathan, and M. S. Rudner, Topological singularities and general classification of Floquet-Bloch systems, New J. Phys. **17**, 125014 (2015).

[S9] R. X. Zhang, and Z. C. Yang, Theory of Anomalous Floquet Higher-Order Topology: Classification, Characterization, and Bulk-Boundary Correspondence, arXiv. 2010, 07945 (2020).

[S10] W. W. Zhu, Y. D. Chong, and J. B. Gong, Symmetry Analysis of Anomalous Floquet Topological Phases, Phys. Rev. B **104**, L020302 (2021).
